# *Lactobacillus brevis* M2-Fermented Whey Protein Hydrolysate Increases Slow-Wave Sleep via GABA_A_ Receptors in Rodent Models

**DOI:** 10.3390/foods13132049

**Published:** 2024-06-27

**Authors:** Hyowon Lee, Hyeongyeong Kim, Yeok Boo Chang, Kisoo Han, Hyeon-Son Choi, Sung Hee Han, Hyung Joo Suh

**Affiliations:** 1 Department of Integrated Biomedical and Life Science, Graduate School, Korea University, Seoul 02841, Republic of Korea; hyowon2432@gmail.com (H.L.); hyeongyeong999@gmail.com (H.K.); 2 Transdisciplinary Major in Learning Health Systems, Graduate School, Korea University, Seoul 02841, Republic of Korea; oobkoey@gmail.com; 3 Neo Cremar Co., Ltd., Seoul 06142, Republic of Korea; ks.han@cremar.co.kr; 4 Department of Food Nutrition, Sangmyung University, Seoul 03016, Republic of Korea; hsc1970@smu.ac.kr; 5 Institute of Human Behavior & Genetics, Korea University, Seoul 02841, Republic of Korea; sungheeh@korea.ac.kr

**Keywords:** whey protein hydrolysate, *Lactobacillus brevis*, fermentation, GABA, sleep

## Abstract

In this study, we investigated the effects of whey protein hydrolysate (WPH) fermented with *Lactobacillus brevis* on sleep behavior and GABAergic mechanisms in rodent models. Fermentation converted the glutamate in WPH to high (3.15 ± 0.21 mg/mL) levels of γ-aminobutyric acid (GABA). Fermented WPH (WP-SF) enhanced sleep duration in mice by increasing GABA content in the brain. The increase in sleep duration induced by WP-SF resulted from an increase in delta wave activity during non-rapid eye movement sleep, and its sleep-promoting effect in a caffeine-induced insomnia model was characterized by an increase in delta waves. WP-SF increased GABAergic receptors at both mRNA and protein levels. Cotreatment with GABA_A_ receptor antagonists abolished the sleep-promoting effects of WP-SF, indicating that WP-SF shares binding sites with antagonists on GABA_A_ receptors. Collectively, WP-SF effectively increased sleep duration by enhancing delta wave activity through GABAergic activation; thus, it is suggested as a functional food-grade ingredient for promoting sleep.

## 1. Introduction

Sleep plays an important role in relieving physical and mental fatigue and is essential for maintaining health. However, the number of people experiencing sleep disorders is increasing, and more than 20% of the population has sleep disorders [1]. The main causes of sleep disorders are diverse and include increased stress, aging, substance abuse, and changes in the sleep cycle; the frequency of sleep disorders owing to stress is high. Sleep-inducing aids, such as serotonin (5-hydroxytryptamine), melatonin, and γ-aminobutyric acid (GABA), have been used to treat these disorders [2]. Benzodiazepines (BDZ), non-benzodiazepines, and antidepressants [3] are among the drugs currently used to treat sleep disturbances. BDZs enhance the inhibitory action of GABA by increasing the chloride ion permeability across cell membranes during nerve stimulation. They act on GABA receptors and increase the number of chloride (Cl)-ion channel openings by GABA, and have been reported to induce sedative and sleepy effects depending on the dose used [4]. Among non-BDZ sleeping pills, the recently developed zolpidem, zaleplon, and eszopiclone exert sleep-inducing effects on sleep through GABA [5]. However, because these drugs have many side effects, such as tolerance and dependence, when used for a long time, natural product-driven ingredients that can improve insomnia with fewer side effects are required [6,7].

Protein hydrolysates are proteins produced by breaking down whole proteins into smaller peptide fragments and free amino acids through hydrolysis [8]. Peptides, with structures similar to naturally occurring substances in the body, exert less influence on immune responses and metabolic processes, thus posing fewer safety concerns [8]. Recent studies have demonstrated the use of protein hydrolysates or peptides as complementary interventions in managing various disease conditions such as hypertension, diabetes, and anxiety [9]. These physiologically active peptides are obtained through enzymatic processes or microbial fermentation from food sources like whey protein [9]. Additionally, synthetic peptides have been developed for therapeutic and cosmetic applications [9,10]. Whey protein hydrolysates, comprising high-quality amino acids and a variety of peptide sizes, are widely recognized for their bioactive compounds and functional properties [11]. Particularly, whey protein hydrolysates offer a range of benefits, catering to athletes, individuals with specific nutritional needs, and those with digestive issues [12]. Recent studies report that whey protein hydrolysates modulate sleep behaviors [13,14,15].

GABA, an inhibitory neurotransmitter, is mainly used to treat sleep disturbances [16,17]. An increase in GABA levels results in emotional stability and anti-anxiety and anticonvulsant effects [18]. Whey protein contains approximately 15–16% glutamic acid, a precursor to GABA, and protease hydrolysis increases the glutamate (Glu) content [19]. The Glu contained in whey protein hydrolysate (WPH) is an excellent natural material that can produce GABA through fermentation [20]. GABA not only exists in animals and plants, but is also a metabolite of microorganisms [21,22] and is produced from Glu via decarboxylation by glutamate decarboxylase [23]. GABA is in the spotlight as a functional food ingredient because of its various effects such as nerve inhibition, mental stability, lowering of blood pressure, and promotion of brain metabolism.

Many food ingredients contain very little GABA content; hence, exhibiting beneficial effects is difficult [24]. GABA is produced by the glutamate decarboxylase of lactic acid bacteria (LABs), which produces high concentrations of GABA. LABs include *Lactobacillus, Leuconostoc, Pediococcus, Lactococcus,* and *Streptococcus* [21]. In this study, fermented whey protein hydrolysate (WP-SF) prepared using the *Lactobacillus brevis* M2 strain was investigated for its effects on sleep behaviors through pentobarbital-induced sleep and electroencephalogram (EEG) analyses in a rodent model, including a caffeine-induced sleep disturbance condition. Additionally, this study investigated the receptor binding mode, with a particular focus on GABAergic regulation.

## 2. Materials and Methods

### 2.1. Chemicals

GABA, l-glutamate, picrotoxin (PIX), bicuculline (BIC), and flumazenil (FMZ) were purchased from Sigma-Aldrich (St. Louis, MO, USA). d-glucose was procured from Duksan Pure Chemicals (Ansan, Republic of Korea).

### 2.2. Fermentation of Whey Protein Hydrolysate (WPH)

WPH was obtained from Neocrema (Seoul, Republic of Korea), where it was prepared by a previously described method [25]. The provided WPH was dissolved at 5% in distilled water, 2% each of Glu and glucose added, autoclaved (121 °C, 103.4 Pa, 15 min), and allowed to cool to room temperature. Thereafter, 4% *L. brevis* M2 was added and incubated at 37 °C for 0–96 h. Following incubation, the supernatant was recovered by centrifugation (4 °C, 4000 rpm, 20 min) and lyophilized to obtain WPH fermentation product (WP-SF). *L. brevis* M2 was pre-cultured at 37 °C for 24 h in de Man, Rogosa, and Sharpe (MRS) broth.

### 2.3. Assay of Total Sugar

The total sugar content of WP-SF was estimated using the phenol–sulfuric acid method [26]. One mL of 5% phenol (*w*/*v*) and 5 mL of sulfuric acid were reacted with 1 mL of sample, following which the absorbance was measured at 490 nm. d-glucose was used as the standard.

### 2.4. Analysis of Glu and GABA by High-Performance Liquid Chromatography (HPLC)

Glu and GABA analysis was performed according to the AccQ-tag method [23] using HPLC equipped with a quaternary solvent delivery system (Waters Alliance e2695 separation module, Waters Corporation, Milford, MA, USA), an autosampler, and a fluorescence detector (Waters Alliance, e2475).

### 2.5. Experimental Animals

Specific pathogen-free ICR mice (male, 25–30 g, 5 weeks old) were purchased from Oriental Bio (Seongnam, Republic of Korea), and SD rats (male, 200–220 g, 5 weeks old) were obtained from Daehan Biolink (Eumseong, Republic of Korea). The environmental temperature of the animal breeding room was 20 ± 2 °C, with a relative humidity of 50 ± 55%, and the light and dark conditions were controlled at a 12 h cycle. Drinking water and food (Feed Lab Korea, Guri, Republic of Korea) were provided ad libitum. The animal experiments were approved by the Korea University Animal Experiment Ethics Committee (KUIACUC-2022–0090, approval date: 21 November 2022).

### 2.6. Evaluation of Sleep Latency and Duration in Pentobarbital-Treated Mice

The ICR mice were deprived for 24 h prior to the experiment and tested according to a previous experimental method [27] between 1:00 PM and 6:00 PM. The samples were administered orally (p.o.), and 40 min later, pentobarbital (42 mg/kg) was injected intraperitoneally (i.p.) to measure sleep latency and duration. Animals in which sleep was not induced within 15 min of pentobarbital administration were excluded. In the caffeine-induced insomnia model, caffeine (40 mg/kg) was orally administered before sample administration.

### 2.7. EEG Analysis in SD Rats

Electrode (Emka Technologies, Paris, France) attachment and EEG analysis were performed in the same manner as described previously [28]. The rats were randomly assigned to six groups (n = 6/group): normal control (NOR, water), positive (BDZ, 200 μg/kg), low dose of WP-SF (WP-SFL, 100 mg/kg), and high dose of WP-SF (WP-SFH, 150 mg/kg). EEG measurements were conducted daily for six consecutive days between 11:00 and 18:00. In the caffeine-induced sleep disturbance model, caffeine (40 mg/kg) was orally administered before sample administration.

### 2.8. GABA_A_ Receptor Binding Test with Antagonists

To evaluate the GABA mechanism related to sleep promotion by WP-SF, the GABA_A_ receptor antagonists PIX (4 mg/kg), BIC (6 mg/kg), and FMZ (10 mg/kg) were used. Each antagonist was dissolved in soybean oil (5% dimethyl sulfoxide) and injected intraperitoneally. WP-SFH or water was administered orally 20 min after the administration of the antagonist, and sleep latency and duration were measured in the pentobarbital sleep induction model as described above.

### 2.9. Analysis of Gene Expression Levels of GABAergic and Serotonergic Receptors Using SYBR™ Green qRT-PCR

ICR mice were administered WP-SF (WP-SFL: 100 mg/kg; WP-SFH: 150 mg/kg) orally five times a week for 3 weeks. Brain tissues were collected post-sacrifice. RNA was extracted using TRIzol reagent (Invitrogen, Carlsbad, CA, USA) from the brain tissue. cDNA was synthesized from 1 μg of total RNA with Superscript^®^ III Reverse Transcriptase (Invitrogen). cDNA (100 ng) was amplified using the StepOne Plus Real-Time PCR System (Thermo Fisher Scientific, Waltham, MA, USA) with Power SYBR™ Green PCR master mix (Thermo Fisher Scientific). Gene expression levels were normalized to GAPDH and calculated using the delta–delta Ct method. Primer details are listed in Table S1.

### 2.10. Analysis of Protein Levels of GABAergic and Serotonergic Receptors Using Western Blotting

The supernatant from homogenized brain tissue was lysed and 40 μg of protein was separated on 10% SDS-PAGE, then transferred to an Immobilon-P PVDF membrane (Millipore, Burlington, MA, USA). The membrane was blocked with 5% BSA, incubated with primary antibodies overnight at 4 °C, followed by goat anti-rabbit IgG-HRP (1:2000, Cell Signaling Technology, Danvers, MA, USA) for 2 h at room temperature. After three washes with TBST buffer (Dynebio Inc., Seongnam, Republic of Korea), the membrane was visualized using Clarity ECL (Bio-Rad, Hercules, CA, USA) on a UVP GelStudio system (Analytik Jena AG, Jena, Germany). Primary antibodies: anti-GABAA receptor (Abcam, Cambridge, UK; 1:1000), anti-5HT1A (Abcam; 1:1000), anti-GABAB receptor (Cell Signaling Technology; 1:1000), anti-β-actin (Cell Signaling Technology; 1:1000), and goat anti-rabbit IgG-HRP (Cell Signaling Technology; 1:2000).

### 2.11. Statistical Analysis

Data were analyzed using SPSS (version 26.0; IBM, Chicago, IL, USA). In vivo and in vitro data are presented as mean ± SEM and SD, respectively. Group differences were assessed by one-way ANOVA with Tukey’s test for significance (*p* < 0.05).

## 3. Results

### 3.1. GABA Production by Lactobacillus brevis M2

As fermentation time increased, the pH decreased from 6.79 to 4.63, turbidity increased from 1.05 to 1.28, and the content of total sugars decreased from 17.45 to 4.85 mg/mL (Table 1). Additionally, L. brevis M2 led to a decrease in Glu content (from 19.33 to 8.55 mg/mL) with a simultaneous increase in GABA levels (23.69 μg/mL) following 96 h fermentation. This indicated that bacterial fermentation facilitated the conversion of Glu to GABA in WPH.

### 3.2. Sleep Latency and Duration in Mice Treated with Pentobarbital Following Administration of WP-SF

**Figure 1:**
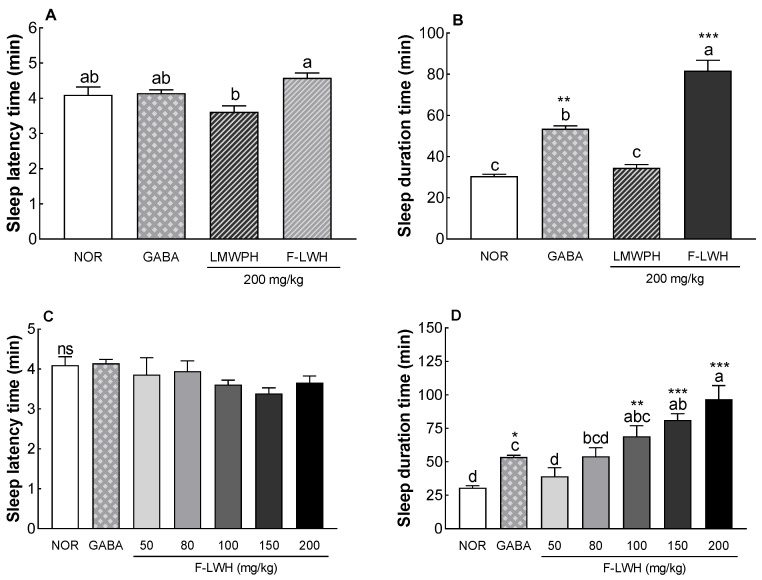
Effects of WPH and WP-SF on sleep latency time (**A**,**C**) and sleep duration (**B**,**D**) in mice that received a hypnotic dose of pentobarbital (42 mg/kg, i.p.). NOR: normal control group (water, p.o.); GABA: γ-aminobutyric acid (80 mg/kg); WPH: whey protein hydrolysate (200 mg/kg, p.o.); WP-SF: fermented WPH (at the indicated doses, p.o.). Different letters indicate significant differences: * *p* < 0.05, ** *p* < 0.01, *** *p* < 0.001 as compared with the NOR group; ns—not significant.

The effects of WPH and WP-SF (200 mg/kg) on sleep latency and duration were measured in ICR mice treated with pentobarbital (Figure 1). A significant increase in sleep duration was observed in the GABA-treated group (80 mg/kg). WPH administration did not result in any change in sleep duration compared to the NOR group, whereas WP-SF significantly increased sleep duration in a dose-dependent manner. Specifically, at a dose of 200 mg/kg, WP-SF increased sleep duration by 2.7-fold. However, sleep latency was not significantly affected by either WPH or WP-SF. These results indicate that fermented WP-SF has sleep-promoting effects.

### 3.3. Changes in EEG during Sleep Owing to WP-SF Administration

**Figure 2:**
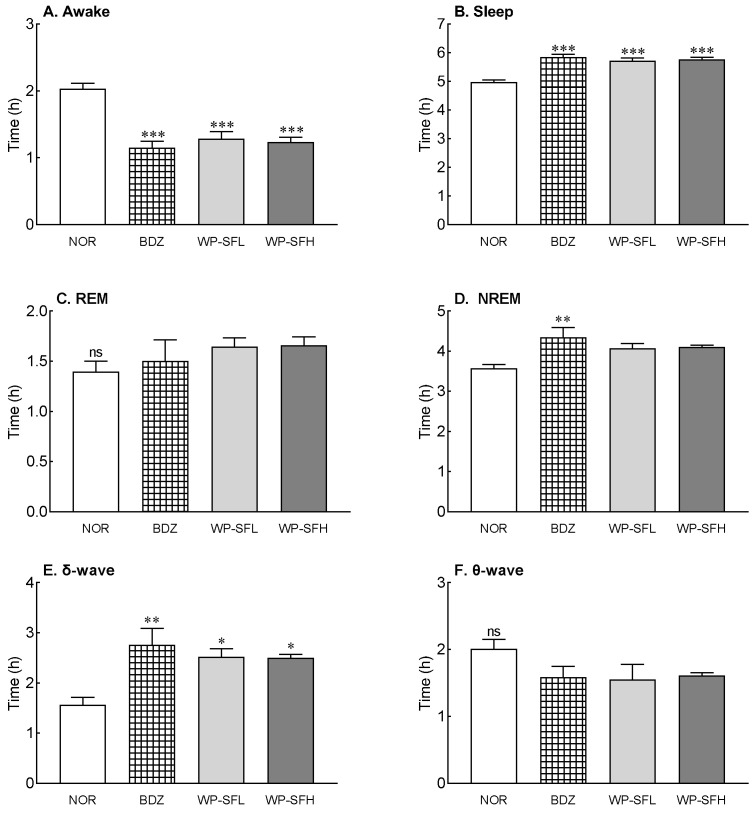
Effects of WP-SF on EEG pattern in rats. EEG analyses were conducted for 6 days. (**A**) Awake time, (**B**) sleep time, (**C**) duration of rapid eye movement (REM), (**D**) duration of non-rapid eye movement (NREM), (**E**) δ-wave of NREM, and (**F**) θ-wave of NREM. NOR: normal control group (water); BDZ: positive control group (benzodiazepine, 200 μg/kg); WP-SF: fermented whey protein hydrolysate; WP-SFL: WP-SF, 100 mg/kg; WP-SFH: WP-SF, 150 mg/kg. * *p* < 0.05, ** *p* < 0.01, *** *p* < 0.001 as compared with the NOR group; ns—not significant.

To analyze the changes in sleep patterns following WP-SF administration, EEG analysis was performed on SD rats (Figure 2). As shown in Figure 2, WP-SF administration significantly increased sleep time and reduced waking time. WP-SFH increased sleep time by 16.1% compared to the NOR group, a level similar to that of BDZ, which was used as a positive control. Although WP-SF administration tended to increase both REM and NREM sleep (Figure 2C,D), these changes were not statistically significant. Specifically, WP-SFL and WP-SFH significantly increased the δ-wave time during NREM, with a 60–62% increase observed as compared with the NOR group (*p* < 0.05; Figure 2E). However, the duration of θ-waves was not significantly altered by WP-SF administration. This result suggests that the WP-SF-mediated increase in sleep duration is attributable to the enhancement in δ-wave activity during NREM sleep.

### 3.4. Insomnia-Alleviating Effects of WP-SF in a Caffeine-Induced Insomnia Mouse Model

**Figure 3:**
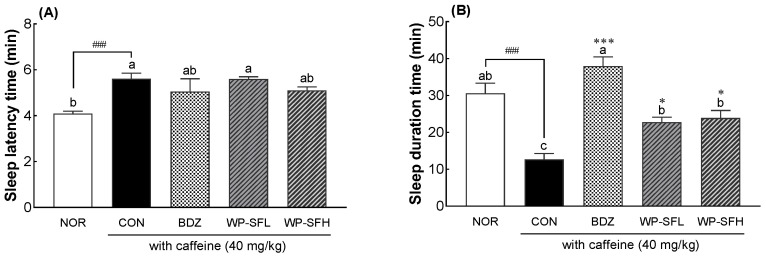
Effects of WP-SF on sleep latency time (**A**) and sleep duration (**B**) in mice that received a hypnotic dose of pentobarbital (42 mg/kg, i.p.). NOR: normal control group (water), CON: caffeine control group (caffeine, 40 mg/kg), BDZ: positive control group (benzodiazepine, 200 μg/kg), WP-SF: fermented whey protein hydrolysate, WP-SFL: WP-SF, 100 mg/kg; WP-SFH: WP-SF, 150 mg/kg. Different letters indicate significant differences: ^###^ *p* < 0.001 as compared with the NOR group; * *p* < 0.05, *** *p* < 0.001 as compared with the CON group.

The insomnia-alleviating effects of WP-SF were evaluated in the caffeine-treated mice (Figure 3). Administration of caffeine significantly increased sleep latency in the CON group compared to the NOR group (*p* < 0.001, Figure 3A), whereas sleep duration decreased significantly (*p* < 0.001, Figure 3B). Thus, caffeine administration effectively induces insomnia. Furthermore, sleep latency tended to decrease after BDZ and WP-SFH administration; however, this difference was not statistically significant (Figure 3A). WP-SF significantly reversed the caffeine-induced decrease in sleep duration. WP-SFL and WP-SFH increased sleep duration by 79% and 89%, respectively, compared with the CON group. BDZ administration resulted in a threefold increase in sleep duration compared to the CON group (*p* < 0.001; Figure 3B). These results suggest that WP-SF ameliorates caffeine-induced insomnia.

**Figure 4:**
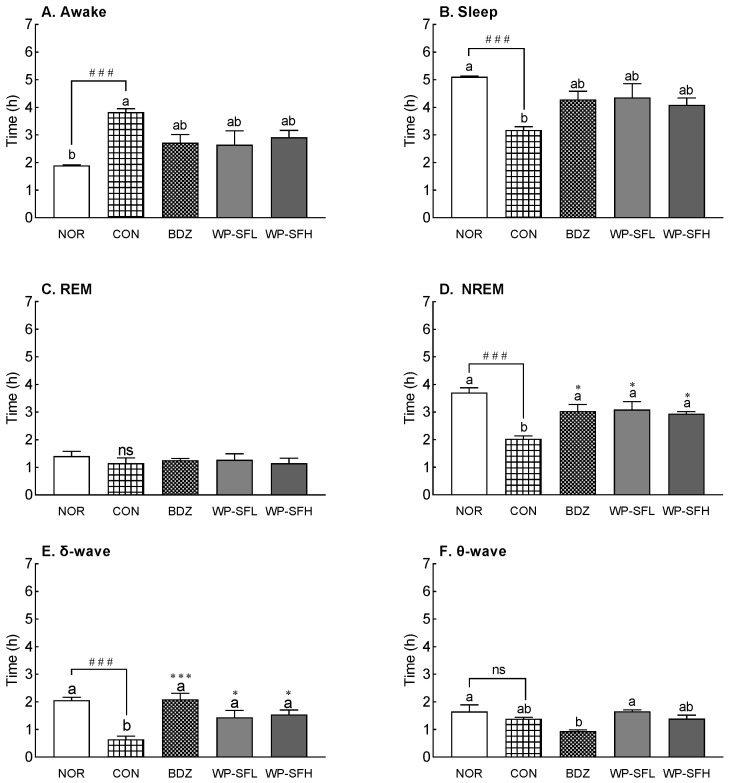
Effects of WP-SF on EEG pattern in rats. EEG analyses were conducted for 4 days. (**A**) Awake time, (**B**) sleep time, (**C**) duration of rapid eye movement (REM), (**D**) duration of non-rapid eye movement (NREM), (**E**) δ-wave of NREM, and (**F**) θ-wave of NREM. NOR: normal control group (water); CON: caffeine control group (caffeine, 40 mg/kg); BDZ: positive control group (benzodiazepine, 200 μg/kg); WP-SF: fermented whey protein hydrolysate; WP-SFL: WP-SF, 100 mg/kg; WP-SFH: WP-SF, 150 mg/kg. To induce an insomnia model, all groups except the normal group were orally administered 40 mg/kg of caffeine. Different letters indicate significant differences: ^###^ *p* < 0.001 as compared with the NOR group; * *p* < 0.05, *** *p* < 0.001 as compared with the CON group, ns—not significant.

After insomnia was induced in the rats by caffeine administration, EEG analysis was performed (Figure 4). Sleep pattern analysis showed that caffeine administration resulted in a significant decrease in both total sleep time and NREM sleep, with reductions of 37% and 45%, respectively, compared with the NOR group. In particular, δ-wave sleep during NREM decreased by 68% with caffeine administration. Although both WP-SF and BDZ increased sleep duration, NREM, and δ-wave sleep patterns, the change in sleep duration was not statistically significant. Instead, both WP-SF and BDZ significantly increased NREM sleep and δ-wave activity. WP-SF showed an increase in NREM sleep, similar to that observed in the BDZ group. δ-wave duration in both WP-SF and BDZ groups increased by 2.1–2.3- and 3.2-fold, respectively, compared with the CON group. These observations indicated that WP-SF effectively counteracted the caffeine-induced decrease in NREM sleep and δ-wave activity.

### 3.5. GABA Content and GABA Receptor Expression Levels in the Brain of Mice after 3 Weeks of Oral Administration of WP-SF

**Figure 5:**
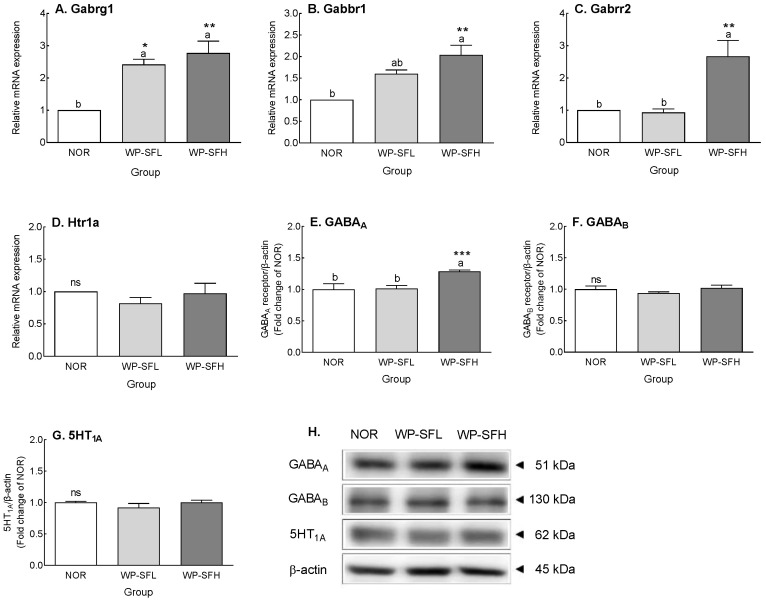
Effects of WP-SF on mRNA and protein expression levels of GABAergic and serotonergic receptors in mice. mRNA level of Gabrg 1, Gabbr1, Gbrr2, and Htr1a was determined by real-time PCR (**A**–**D**). Protein level of GABA A/B and 5HT1Awas examined using Western blot (**E**–**H**). NOR: normal control group (water); WP-SF: fermented whey protein hydrolysate; WP-SFL: WP-SF, 100 mg/kg; WP-SFH: WP-SF, 150 mg/kg. Different letters indicate significant differences: * *p* < 0.05, ** *p* < 0.01, and *** *p* < 0.001 vs. NOR, ns—not significant. Gabrg1: GABAA receptor subunit gamma 1; Gabbr1: GABAB receptor subunit 1; Gabrr2: GABAA receptor subunit rho2; Htr1a: 5-hydroxytryptamine receptor 1A; 5HT1A: 5-HT1A receptor.

GABA content (Table 2) and receptor expression (Figure 5) in the brain were analyzed after oral administration of WP-SF to mice for 3 weeks. WP-SFL did not alter the GABA level in the brain, whereas WP-SFH led to a significant increase of approximately 21.161 μg/mg, which was significantly higher than that of NOR (*p* < 0.05, Table 2). WP-SFL administration significantly increased the expression of GABA receptors (*p* < 0.001; Figure 5A,B). WP-SF administration increased Gabrg1 and Gabbr1 by 2.4–2.8-fold and 1.6–2.0-fold, respectively, compared to the NOR group. Additionally, a high dose of WP-SFH increased gene expression of Gabrr2 by 2.7-fold, compared to that in the NOR group. However, the effect of WP-SF on Htr1a mRNA expression was not significant. At the protein level, only the GABA_A_ receptor level significantly increased (35%) after WP-SFH administration (*p* < 0.001; Figure 5E). Protein levels of the GABA_B_ receptor and 5HT_1A_ were not significantly altered by WP-SF. These results indicate that WP-SF significantly enhanced GABA levels and GABA_A_ receptor expression in the mouse brain.

### 3.6. Binding Site of WP-SF in the GABA_A_ Receptor

**Figure 6:**
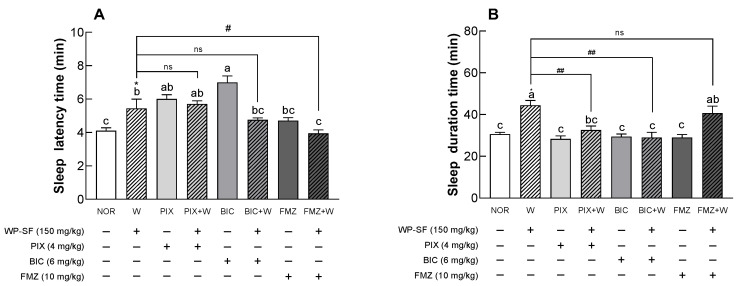
Effects of GABA_A_ receptor antagonist on sleep latency time (**A**) and sleep duration (**B**) in mice that received a hypnotic dose of pentobarbital (42 mg/kg, i.p.). NOR: normal control group (water); WP-SF: fermented whey protein hydrolysate; W: WP-SF, 150 mg/kg; PIX: picrotoxin (4 mg/kg); BIC: bicuculline (6 mg/kg); FMZ: flumazenil (10 mg/kg). Different letters indicate significant differences: * *p* < 0.05 compared to the NOR group; ^#^ *p* < 0.05; ^##^ *p* < 0.01 between the antagonist treatment and non-antagonist treatment (WP-SF group), ns—not significant.

To identify the binding site in the GABA_A_ receptor on which WP-SF acts, changes in sleep duration were measured using GABA_A_ receptor antagonists (PIX, BIC, and FMZ). When each antagonist and WP-SF were administered, PIX, BIC, and FMZ significantly reduced sleep duration compared to WP-SF alone (*p* < 0.01; Figure 6B). Co-administration of PIX or BIC with WP-SF did not significantly alter sleep duration, whereas co-administration of FMZ and WP-SF significantly increased sleep duration. These results indicated that WP-SF shared binding sites with PIX and BIC on the GABA receptor, whereas FMZ and WP-SF stimulated different sites on this receptor. These findings indicate that the WP-SF-mediated increase in sleep duration was associated with the activation of PIX or BIC sites on the GABA_A_ receptor.

## 4. Discussion

GABA is an inhibitory neurotransmitter in the CNS, constituting 30–40% of all neurotransmitters [13], with concentrations 200–1000 times higher than other neurotransmitters. Elevated GABA levels promote emotional stability, and exert anti-anxiety, anticonvulsant, and sleep-inducing effects [18]. Natural GABA content is minimal and degrades with heat during food processing, limiting its physiological efficacy. Fermentation with microorganisms has been studied to enhance GABA content in natural ingredients [29]. Whey protein hydrolysates, rich in Glu, serve as substrates for GABA production.

LAB is used to produce GABA [21,22,30], which is produced by the irreversible decarboxylation of Glu by glutamic acid decarboxylase (GAD), and GAD and GABA are found in higher organisms and microorganisms. The levels of the excitatory neurotransmitter Glu and the inhibitory neurotransmitter GABA are regulated by GAD [31]. Before fermentation (0 h), the Glu content was 19.33 mg/mL, and no decrease in the formation of Maillard reaction products due to heat treatment was observed. Maillard reaction products are produced by a chemical reaction at high temperature between the amino group of a protein and the carbonyl group of a reducing sugar. In the fructose and glucose amino acid reaction model, when heated at 130 °C for 2 h, Glu showed little browning due to the Maillard reaction, and among 20 amino acids, MRP was rarely generated by Glu [32]. The whey protein hydrolysate used in this study did not show a decrease in Glu content due to the autoclave conditions prior to inoculation with the fermentation strain (Figure S1).

Fermentation for GABA production was performed using L. brevis M2, a heterofermentative strain that harbors the gadA and gadB genes encoding glutamate decarboxylase [33]. GABA content increased to 3.15 mg/mL after 96 h fermentation. The substrate glutamic acid content initially decreased until 72 h, after which it increased at 96 h (Table 1). This pattern was also observed in soymilk hydrolysates fermented with *L. plantarum* [34], likely due to glutaminase activity. LAB strains such as *L. brevis* HP2, L. fermentum HP3, and *L. plantarum* MNZ are known to elevate Glu levels [35].

The increase in GABA (Table 1) may partly contribute to the sleep-promoting effect of WP-SF, which exhibited a longer sleep duration compared to WPH (Figure 1). Previous studies have reported the sleep-enhancing effects of GABA-containing foods. Rice germ extract containing GABA alleviates insomnia [36], and GABA-rich fermented milk decreases sleep latency and increases sleep time in patients [37]. However, GABA-containing foods may not consistently promote sleep solely based on their GABA content. The GABA content in WPH at 100 mg, calculated to be 3.15 mg, may not be sufficient to induce sleep promotion, as previous research suggests effective GABA levels in the range of 60–100 mg/kg [38]. Thus, other components in WPH, such as peptides, may promote sleep. Peptides like DIQK and VPFF are potential active components [13] in that their effects are enhanced with GABA. Additionally, peptides like YLGYLEQLIR, YPVEPF, and YFYPEL have been reported to enhance sleep [14,15]. In WPH, hydrolyzed by enzyme and fermentation, various DIQK- or VPFF-like peptides likely contribute to sleep promotion, with GABA playing a supporting role. Further research is needed to isolate and purify the sleep-promoting peptides in WP-SF.

WP-SF enhanced sleep time and mitigated insomnia in a caffeine-induced model. Caffeine enhances alertness, activates the cortex, lessens fatigue, and impedes adenosine receptors, inducing wakefulness and diminishing cortical slow-wave activity [39,40]. Brain GABA content diminishes with increasing caffeine doses [41]. GABA-containing extracts improve sleep disturbances caused by caffeine [36]. WP-SF, containing GABA, improved insomnia resulting from caffeine intake. The enhancement in sleep duration and insomnia relief led to prolonged NREM sleep, owing to the surge in δ-waves instigated by WP-SF (Figure 2). Sleep is classified into REM and NREM. Intellectual functions, including memory, are restored during REM sleep. NREM sleep, constituting 75% of sleep, facilitates physical restoration and comprises shallow and deep stages. Delta-wave sleep (slow-wave), signifying the deep stage, predominantly occurs in the initial third of the cycle, while REM sleep is prevalent in the final third [42]. WP-SF, exhibiting an increase in delta waves, is associated with deep sleep induction.

Many natural products known for their sleep-improving effects have been reported to increase sleep duration and NREM sleep via the GABA_A_ receptors [28,43]. This implies that the increase in gene expression and protein levels of GABA_A_ receptors after oral administration of WP-SF for 3 weeks resulted from the involvement of these receptors, resulting in increased sleep time (Figure 5). Among the GABA receptors, GABA_A_, the ionotropic receptor that regulates the opening and closing of Cl^−^ channels, plays important roles in sedation, sleep, and anesthesia [44]. The increased penetration of Cl^−^ ions into postsynaptic cells strengthens the inhibitory effect of GABAergic neurotransmission, resulting in sleep activity [45,46]. Thus, the increase in GABA_A_ receptor gene and protein expression caused by WP-SF administration promotes the influx of Cl^−^ ions into the cells through GABA_A_ receptors, thereby increasing sleep time. WP-SF extended sleep duration by binding to the BIC and PIX binding sites on GABA_A_ receptors (Figure 6). GABA_A_ receptors have binding sites for GABA, barbiturates, picrotoxins, steroids, and benzodiazepines [47,48]. Although alcohol can bind to the GABA_A_ receptors, limited information is available regarding the exact binding sites. GABA responses are inhibited by BIC or PIX, which competitively or non-competitively block GABA_A_ receptors. BIC, a competitive antagonist of GABA_A_ receptors, regulates Cl^−^ ion channels. In contrast, PIX, a non-competitive antagonist of GABA_A_ receptors, acts directly on the GABA_A_ receptor channels involved in the passage of Cl^−^ ions [49]. Therefore, BIC and PIX inhibit the permeability of Cl^−^ ions, promoting their inhibitory influence on target neurons [50]. FMZ, an antagonist that blocks sleep activity by binding to benzodiazepine receptors, reduces all brain wave ranges except the gamma band caused by sleeping pills, thereby simultaneously inducing awakening [51]. The observed reduction in sleep time (Figure 6) owing to PIX and BIC suggests that WP-SF modulates the influx of Cl^−^ ions through competitive and non-competitive actions on GABA_A_ receptors.

## 5. Conclusions

In this study, to improve the sleep-inducing activity of WPH, a hydrolysate of Alcalase, Flavourzyme, and Protamex, the GABA content was increased through fermentation with *Lactobacillus brevis* M2. WP-SF, with the increase in GABA, extended total sleep time and enhanced the NREM sleep pattern. This effect was corroborated using a caffeine model. The sleep-inducing effect mediated by WP-SF was achieved through the upregulation of GABA content and GABA_A_ receptor expression in the mouse brain. Furthermore, WP-SF interacted with the GABA binding site on GABA_A_ receptors, binding to PIX or BIC, which are GABA_A_ receptor antagonists. These results suggest the potential applications of WP-SF, with a focus on sleep-promoting functions of fermented whey protein hydrolysates. However, GABA did not independently explain the sleep characteristics observed in WP-SF. Thus, further studies on other potentially active peptides are needed.

## Figures and Tables

**Table 1:** Changes in composition during WPH fermentation by *Lactobacillus brevis* M2.

Time (h)	pH	Absorbance (600 nm)	Total Sugar (mg/mL)	Glu (mg/mL)	GABA (mg/mL)
0	6.79	1.051 ± 0.037	17.45 ± 0.23 ^a^	19.33 ± 0.30 ^a^	0.14 ± 0.05 ^d^
12	6.46	1.060 ± 0.002	16.71 ± 0.03 ^b,^***	-	-
24	6.09	1.093 ± 0.005	15.21 ± 0.05 ^c,^***	-	-
48	4.87	1.245 ± 0.020	6.76 ± 0.14 ^d,^***	16.26 ± 0.19 ^b,^***	1.68 ± 0.09 ^c,^***
72	4.64	1.261 ± 0.006	4.85 ± 0.08 ^e,^***	8.55 ± 0.09 ^c,^***	2.18 ± 0.003 ^b,^***
96	4.63	1.281 ± 0.013	4.29 ± 0.04 ^f,^***	14.87 ± 0.07 ^b,^***	3.15 ± 0.21 ^a,^***

Different letters indicate significant differences: *** *p* < 0.001 compared with the NOR group, based on one-way ANOVA and Tukey’s multiple comparison test.

**Table 2:** GABA content in mouse brains after 3 weeks of oral administration of WP-SF.

Sample	GABA (μg/mg of Sample)
NOR	17.50 ± 0.26 ^b^
WP-SFL	16.22 ± 0.81 ^b^
WP-SFH	21.16 ± 0.48 ^a^*

Different letters indicate significant differences * *p* < 0.05, compared to the NOR group. GABA: γ-aminobutyric acid; WP-SP: fermented whey protein hydrolysate; NOR: normal control group (water); WP-SFL: WP-SF (100 mg/kg); WP-SFH: WP-SF (150 mg/kg).

## Data Availability

The original contributions presented in the study are included in the article/Supplementary Materials, further inquiries can be directed to the corresponding author.

## Supplementary Materials

The following supporting information can be downloaded at: https://www.mdpi.com/article/10.3390/foods13132049/s1, Table S1. PCR primer sequences. Figure S1. Glu analysis of whey protein hydrolysates before and after autoclaving using HPLC.
