# Supplementary material for: Lactobacillus brevis M2-Fermented Whey Protein Hydrolysate Increases Slow-Wave Sleep via GABAA Receptors in Rodent Models
Source: Foods. 2024 Jun 27;13(13):2049. doi: 10.3390/foods13132049 (PMC11241806; doi:10.3390/foods13132049)
Supplement: Supplementary file 1 [file foods-13-02049-s001.zip › foods-2992931-supplementary.pdf]

Table S1. PCR primer sequences

Target gene		5′-sequence-3′	NCBI Reference Sequence
Gabrg1	Forword	CTACCAGTGTTTGGAGGGCAA	NM_001417487.1
	Reverse	GTATGTGTATCCGCCCTTCCC	
Gabbr1	Forword	GCCAAGGAGGAACCAAAGGA	NM_001413996.1
	Reverse	CAGCAGCAGCCCTTTGTAAC	
Gabrr2	Forword	TTTTGCCTGATGGCTCTCGTG	NM_008076.4
	Reverse	CTTGGATGTTTCCAGAAGCCC	
Htr1a	Forword	ATCTCCATCCCGCCTATGCT	NM_008308.5
	Reverse	AAGCGCCGAAAGTCGAGTAG	
GAPDH	Forword	GTGTCCGTCGTGGATCTGAC	NM_001411843.1
	Reverse	AGTCGCAGGAGACAACCTGG	

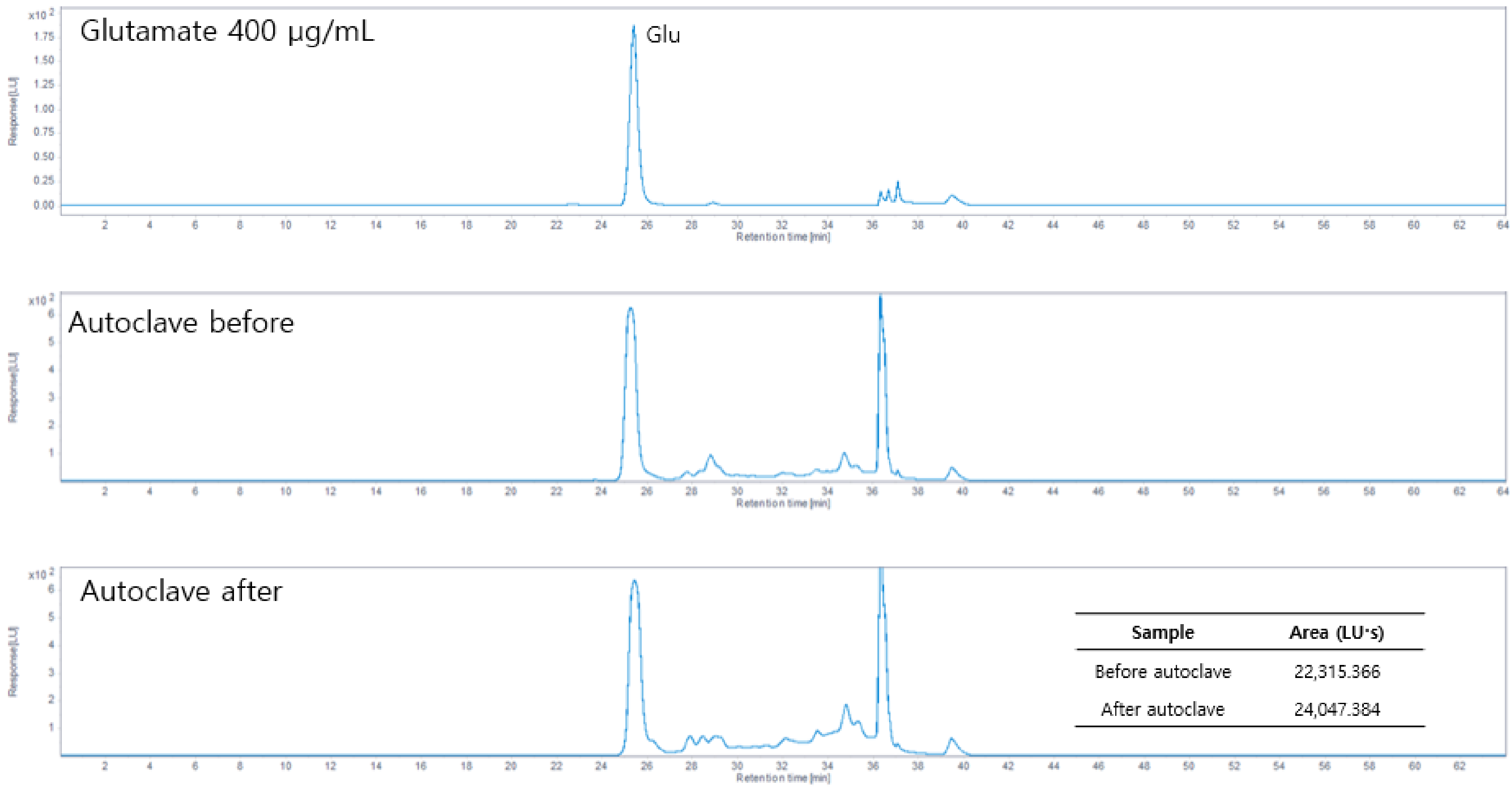

Figure S1. Glu analysis of whey protein hydrolysates before and after autoclaving using HPLC.
